# Trends in Varicella Burden of Disease Following Introduction of Routine Childhood Varicella Vaccination in Argentina: A 12-Year Time Series Analysis

**DOI:** 10.3390/vaccines10071151

**Published:** 2022-07-20

**Authors:** Norberto Giglio, Pieralessandro Lasalvia, Manjiri Pawaskar, Cintia I. Parellada, Yaneth Gil Rojas, Paula Micone, Virginia V. Babic, Silvina E. Neyro

**Affiliations:** 1 Hospital de Niños Ricardo Gutiérrez, Buenos Aires C1425EFD, Argentina; ngiglio@austral.edu.ar; 2 NeuroEconomix, Bogotá D.C. 110831, Colombia; yanekgil@gmail.com; 3 Merck & Co., Inc., Vaccines, Rahway, NJ 19454, USA; manjiri.pawaskar@merck.com; 4 MSD Brazil, São Paulo 04583-110, Brazil; cintia.parellada@merck.com; 5 Hospital Carlos G Durand, Obstetrics, Buenos Aires C1405DCS, Argentina; paulamicone@gmail.com; 6 MSD Argentina, Buenos Aires B1605AZE, Argentina; virginia.verdaguer.babic@merck.com; 7 Ministerio de Salud de la Nación, Dirección de Control de Enfermedades Inmunoprevenibles (DiCEI), Buenos Aires C1073ABA, Argentina; sneyro@dicei.msal.gov.ar

**Keywords:** varicella vaccination, infectious disease burden, surveillance, time series, ARIMA

## Abstract

One-dose universal varicella vaccination (UVV) was introduced in the Argentinian National Immunization Program in July 2015. This study examined the impact of one-dose UVV on varicella incidence and mortality in Argentina. Incidence and mortality data were obtained from official databases for pre-UVV (January 2008–June 2015) and post-UVV (July 2015–December 2019) periods. Time series analyses with autoregressive integrated moving average (ARIMA) modeling predicted varicella incidence and mortality in absence of UVV in the target (aged 1–4 years) and overall population. Predicted and observed values post-UVV were compared to estimate UVV impact. Mean annual incidence rates per 100,000 reduced from 1999 (pre-UVV) to 1122 (post-UVV) in the target population and from 178 to 154 in the overall population. Significant declines in incidence were observed, reaching reductions of 83.9% (95% prediction interval [PI]: 58.9, 90.0) and 69.1% (95% PI: 23.6, 80.7) in the target and overall populations, respectively, during peak months (September-November) post-UVV. Decreasing trends in mortality rate from 0.4 to 0.2 per 1,000,000 population were observed. Over the last four years, one-dose UVV has significantly reduced varicella burden of disease in Argentina. Continuous efforts to improve vaccination coverage rates and long-term follow-up are needed to better understand the benefits of the UVV program.

## 1. Introduction

Varicella is caused by the varicella-zoster virus (VZV) [1,2]. While it is generally perceived as a mild and self-limiting disease, it is highly contagious with patients requiring supportive care and isolation precautions. However, varicella-associated complications can occur in up to 12% of outpatients and 92.6% of inpatients [3,4,5,6,7,8]. Approximately 5% of patients with varicella who seek medical services will require hospitalization [9]. The most common complications are secondary bacterial infections of the skin and soft tissues, followed by pneumonia and encephalitis [3,4,5,6,7,8].

In countries with temperate climates such as Argentina, varicella is primarily a seasonal childhood disease with most cases occurring in late winter and spring (September to November) [9,10,11]. In regions with tropical and subtropical climates, varicella tends to be more pronounced in adolescents and adults [1]. Before the introduction of universal varicella vaccination (UVV) in Argentina, varicella seroprevalence was reported to be as high as 97.6% among individuals under 20 years old in 2002 [12], with children under 10 years old accounting for the largest proportion of those with new infection [13].

The high socioeconomic impact of varicella in pediatric patients in Argentina during the pre-UVV period has been documented [14]. Between 1997 and 2012, a varicella incidence ranging from 250 to 450 cases per 100,000 population was reported, and 272 deaths were recorded, of which 60% were in children aged under 10 years [15]. The estimated costs per pediatric patient for varicella ranged from United States dollar (USD) 340 for outpatients to USD 3109 for inpatients, amounting to an overall annual cost of approximately 40 million dollars in 2015 (2017 USD) [14].

A live-attenuated Oka/Merck strain varicella vaccine (VARIVAX; Merck & Co., Inc., West Point, PA, USA) was approved in the United States in 1995. It has demonstrated proven long-term safety, efficacy, and effectiveness in preventing varicella and has been available globally for over 25 years [16,17,18]. One dose of single-antigen varicella vaccine has been reported to be 85% effective at preventing any form of varicella [19], with two doses increasing this to 98%, with 100% protection against severe varicella [19]. Despite the proven benefits of varicella vaccination, there are currently only 17 countries in Latin America that have implemented one-dose or two-dose UVV programs, [20,21] and few countries have published post-implementation data on the impact of UVV [22,23,24,25,26].

In Argentina, UVV with a live-attenuated Oka/Merck strain was included in the National Immunization Program in January 2015 and rolled out in July 2015, administered as a single dose to infants at the age of 15 months. In 2022, the program was extended to a two-dose schedule, with the second dose administered at the age of five years [27].

The objective of this study was to examine the trends in varicella burden of disease in Argentina following the implementation of the one-dose UVV program in children aged 1–4 years (target population) and in the overall population.

## 2. Material and Methods

### 2.1. Study Design and Data Sources

A retrospective database study was conducted using time series analyses to assess the impact of UVV on disease incidence and mortality in Argentina. Twelve years of varicella surveillance data were analyzed from two periods: pre-UVV, from January 2008 to June 2015, and post-UVV introduction (post-UVV), from July 2015 to December 2019. Data on varicella cases, collected by the Ministry of Health, were extracted from the Argentinian National Health Surveillance System (SNVS), part of the Integrated Health Information System (SISA) [28,29,30]. Mortality data were obtained from the Health Statistics and Information Department (DEIS) [31,32]. Population data, to calculate varicella incidence, were extracted from the National Institute of Statistics and Census (INDEC) [33]. Vaccination coverage data were obtained from the Directorate of Vaccine-Preventable Disease Control (DiCEI) Ministry of Health reports [34].

### 2.2. Study Population

Although the vaccination was only available to the cohort of children aged 15 months old, as no catch-up vaccination program was implemented in Argentina, the study target population for this analysis was defined as children aged 1–4 years to allow future comparisons as new cohorts of 15-month-olds will be eligible each year post-UVV. Therefore, the proportion of potentially vaccinated children increased cumulatively during the first four years post-implementation as the initial 15-month-old cohorts aged, and new 15-month-old cohorts were added. As such, assuming birth cohorts had the same size and vaccination uptake per year, the proportion of children vaccinated was <25% in 2015; 25–50% in 2016; 50–75% in 2017; 75–100% in 2018 onwards.

The non-target population was defined as individuals aged <1 year and ≥5 years. The overall study population included all age groups and was also stratified by age groups: <1, 1–4, 5–9, 10–14, 15–24, 25–34, 35–44, 45–64, and ≥65 years. Data that did not contain a specification of age group was considered for the overall population analysis. Target and non-target population analyses only considered cases with available age data.

### 2.3. Outcomes and Analytical Methods

Varicella is a mandatory reportable disease in Argentina and is required to be reported on a weekly basis by healthcare workers and laboratories at the local, provincial, and national levels. Reported cases were clinically diagnosed by a health practitioner (qualified doctor or nurse) and reported to SNVS according to the official reporting manual [29].

The mean annual incidence was calculated using total cases and age-specific populations and was expressed per 100,000 population. Data from DEIS and INDEC were used to calculate the varicella mortality rate for each month and year, which were reported as number of deaths per 1,000,000 population.

### 2.4. Analysis

A descriptive analysis of mean monthly and annualized varicella incidence and mortality in the pre- and post-UVV periods was conducted and results summarized. The mean absolute and relative differences were calculated when comparing data from the pre- and post-UVV periods. To avoid issues such as time window selection and the presence of secular trends that could bias this type of analysis and interpretation of results, statistical comparisons (95% confidence intervals [CIs] and *p*-values) were only performed for the time series analysis.

### 2.5. UVV Impact Estimation

A time series analysis was performed using an Autoregressive Integrated Moving Average (ARIMA) model. Several time series models with different parameters were trained and tested for each population group. Final models were selected considering the Akaike information criterion (AIC). The model was used to predict expected varicella cases in the post-UVV period in the absence of vaccination, based on previous trends observed in the pre-UVV period with 95% confidence intervals (CI). These predicted values served as a counterfactual to the observed varicella cases reported in the post-UVV period. The point estimate was calculated by subtracting the mean predicted and the observed values. The 95% prediction interval (PI) was calculated by subtracting the 95% CI of the predicted values with the observed values. These estimations were then used to calculate the number of varicella cases avoided in Argentina since the implementation of UVV between July 2015 and December 2019.

Given the consistent and strong seasonal pattern of varicella surveillance data, the predicted data were generated for the entire year, peak periods (September to November, when the highest numbers of cases occurred) and non-peak periods (rest of the year). UVV impact was calculated by comparing predicted and observed varicella incidence and mortality for the entire year in addition to peak and non-peak periods in each year.

Considering that seasonality is a relevant factor, the ratios between high and low incidence periods in the post-UVV period were described in two ways: (i) dividing the average incidence into peak periods (September to November) by non-peak periods (rest of the year) and (ii) dividing the average incidence in the three months with the highest incidence (September to November) by the average incidence in the three months with the lowest incidence (February to April).

## 3. Results

### 3.1. Vaccination Coverage

In the year one-dose UVV was implemented in Argentina (2015), national vaccination coverage was 44.8%. In the following years, national coverage rose to 74.4%, 76.8%, 81.0%, and 77.6% in 2016, 2017, 2018, and 2019, respectively. Out of twenty-four provinces, only five provinces in Argentina have consistently reported vaccination coverage below 80% since one-dose UVV implementation. By 2019, 16 out of 24 (66.6%) provinces had coverage over 80%, with coverage in the remaining 8 provinces ranging from 69.2% to 79.5% (Table S1).

### 3.2. Descriptive Statistics for Varicella Cases, Incidence, and Mortality

During the 12 years covered by this study (2008–2019), 1,426,433 varicella cases were reported in the overall population. Most cases occurred in the age groups 1–4 years (target population) and 5–9 years, comprising 41% and 36% of all reported cases during the study period, respectively (Table 1).

**Figure 1:**
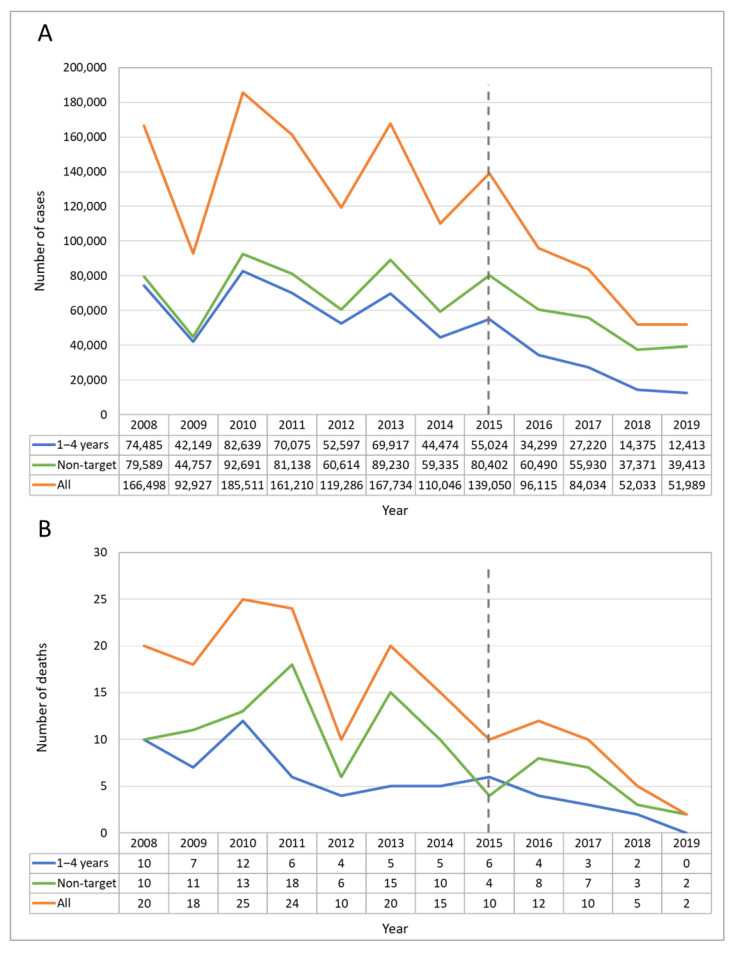
Pattern of reported cases of varicella (**A**) and reported varicella-associated deaths (**B**) in Argentina in the target (1–4 years), non-target (all ages excluding 1–4 years), and overall population, 2008–2019. Overall population data includes target population, non-target population, and cases with no age specification. The dashed line corresponds approximately to the period of UVV introduction in Argentina. UVV, universal varicella vaccination.

In the pre-UVV period, the highest mean annual incidences were reported in those aged 1–4 years (1999 cases per 100,000), followed by <1 year (1326 per 100,000) and 5–9 years (1264 per 100,000). For older age groups, incidence rates ranged from 4.2 (≥65 years) to 261 per 100,000 (10–14 years) in the pre-UVV period (Table 1). The mean annual incidence rate declined across the post-UVV period in all age groups except in children 10–14 years old. The highest mean annual incidence in the post-UVV period was found in those aged 5–9 years (1132 per 100,000), followed by 1–4 years (1122 per 100,000) and <1 year (824 per 100,000). Overall, there was a decreasing trend of reported varicella cases in the target (1–4 years), non-target (all ages excluding 1–4 years), and overall population from 2008 to 2019 (Figure 1A). A total of 171 varicella deaths were reported during the study period. In the pre-UVV period, most deaths (73.5%; n = 100) occurred in the age groups <1, 1–4, and 5–9 years. An overall decline in varicella-associated deaths was observed, from 20 in 2008 to two in 2019, across all age groups (Figure 1B and Table S2). In 2019, no deaths were reported in children aged under five years.

### 3.3. Time Series Analysis

**Figure 2:**
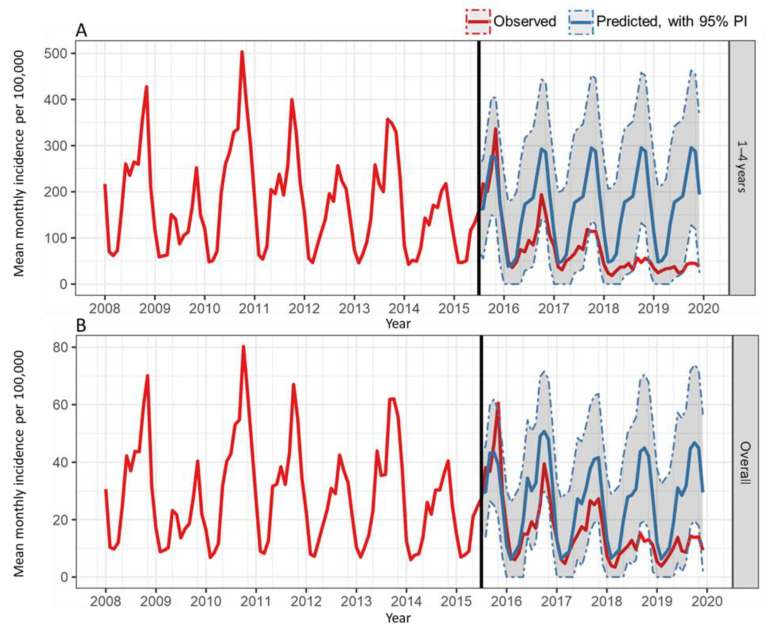
Trends in observed and predicted mean incidences of varicella in the target population (**A**) and overall population (**B**) in the pre- and post-UVV periods, derived from monthly data. The solid line corresponds approximately to the period of UVV introduction in Argentina. CI, confidence interval; observed, observed incidence following UVV introduction; predicted, predicted incidence without UVV introduction; UVV, universal varicella vaccination.

In the post-UVV period, observed mean annual incidence rates were generally lower than the predicted mean annual incidence rates in the absence of UVV for target and overall populations, as shown in Figure 2. Strong seasonal patterns were observed from 2008 to 2016; Figure S1 provides predicted and observed values by age group. Further stratification of incidence rates in peak and non-peak periods showed marked reductions in the follow-up period.

**Figure 3:**
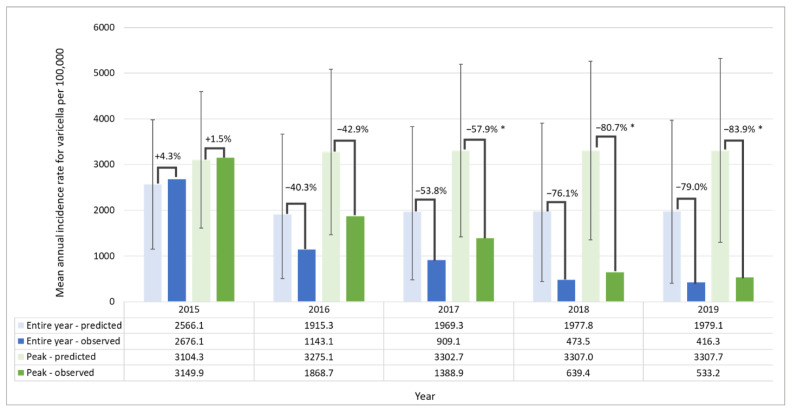
Observed and predicted mean annual incidence rates for varicella in Argentina and relative difference between them, in the entire year and peak periods, in the target population (1–4-year-olds). * Periods where observed values are not within the 95% PI of predicted mean annual incidence rate. Proportion of target population eligible for vaccination: less than 25% in 2015; 25–50% in 2016; 50–75% in 2017; 75–100% in 2018 and 100% in 2019. Vaccination coverage in target population: 44.8% in 2015; 74.4% in 2016; 76.8% in 2017; 81.0% in 2018 and 77.6% in 2019. Non-peak period, all months excluding September to November; observed, observed incidence following UVV introduction; overall period, all months; peak period, September to November; predicted, predicted incidence without UVV introduction; UVV, universal varicella vaccination; PI, prediction intervals.

In the target population, the observed incidence was significantly lower than the predicted incidence (without UVV) in October and November in 2017 (−61.4% and −60.1%), and in September, October, and November in both 2018 (−76.7%, −84.1%, and −80.5%) and 2019 (−82.2%, −84.7%, and −84.5%) (Table S3). In the analysis of peak and non-peak periods, significant differences in observed versus predicted mean incidences were found in peak periods from 2017, reaching reductions of 83.9% (95% PI: 58.9, 90.0) in 2019 (Figure 3). Declines were also observed in non-peak periods from 2016.

**Figure 4:**
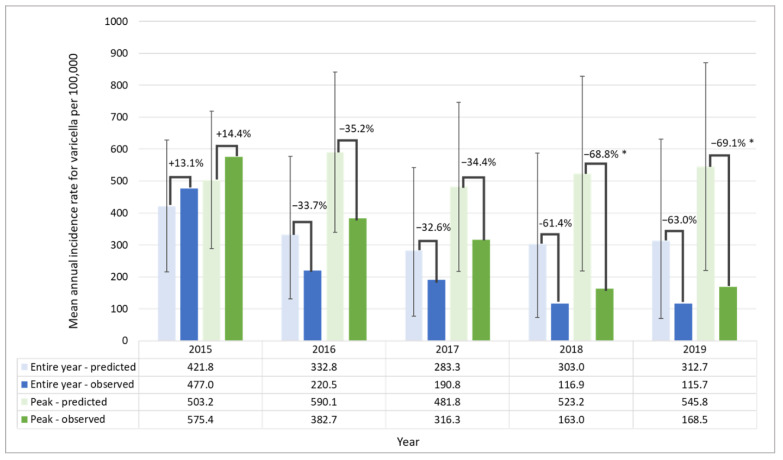
Observed and predicted mean annual incidence rates for varicella in Argentina and relative difference between them, in the entire year and peak periods, in the overall population. * Periods where observed values are not within the 95% PI of predicted mean annual incidence rate. Proportion of target population eligible for vaccination: less than 25% in 2015; 25–50% in 2016; 50–75% in 2017; 75–100% in 2018 and 100% in 2019. Vaccination coverage in target population: 44.8% in 2015; 74.4% in 2016; 76.8% in 2017; 81.0% in 2018 and 77.6% in 2019. Non-peak period, all months excluding September to November; observed, observed incidence following UVV introduction; overall period, all months; peak period, September to November; predicted, predicted incidence without UVV introduction; UVV, universal varicella vaccination; PI, prediction intervals.

In the overall population, there were significant differences between observed and predicted incidence rates for September in 2016 (−48.9%) and for September, October, and November in 2018 (−65.0%, −72.3%, and −69.1%) and 2019 (−68.0%, −70.4% and −68.9%) (Table S4). There was a decreasing trend in the incidence rates from 2015; however, significant reductions were observed in peak periods in 2018 and 2019 (−68.8%, [95% PI: −80.3, −25.4] and −69.1% [95% PI: −80.7, −23.6], respectively; Figure 4). In non-peak periods, reductions from 2016 (−32.6%) to 2019 (−58.3%) were also observed.

The estimated numbers of avoided varicella cases in the post-UVV period are presented in Table S5. Overall, an estimated 144,811 and 249,090 cases were avoided in the target and overall populations, respectively, for the whole post-UVV period. In the analysis considering only peak periods and assuming the remaining months had no differences, significant reductions were observed with an estimated 51,203 (95% PI: 12,152, 90,254) and 92,886 (95% PI: 13,385, 172,388) cases avoided in the target and overall populations, respectively (Table S5).

In the exploratory analysis, comparing average incidence in peak and non-peak periods, rate ratios varied between 2.3 and 1.4 (Table S6). When comparing average incidence in the three highest versus three lowest incidence months, the highest rate ratios were observed in 2015 and they progressively declined each year in the post-UVV period (Table S6).

In the pre-UVV period, the mean annual mortality rate was 0.4 per 1,000,000 population, falling to 0.2 per 1,000,000 population in the post-UVV period (Figure S2). Monthly mortality changes comparing pre- and post-UVV periods are shown in Figure S3.

## 4. Discussion

This study assessed the impact of one-dose UVV following its introduction into the Argentinean National Immunization Program in 2015 by analyzing the burden of varicella in pre- and post-UVV periods using a time series analysis. The greatest reductions in varicella incidence were observed in peak periods (September to December) in the target age group (1–4 years). The indirect benefits of vaccination were also observed in the overall population, including older children and adults not eligible for vaccination.

A total of 249,090 cases of varicella were estimated to be prevented in the overall population from July 2015 to December 2019, of which the majority (144,811) of prevented cases were in children aged 1–4 years. The reported number of varicella cases decreased gradually since the introduction of one-dose UVV.

Vaccination coverage increased over time in Argentina, with a greater number of provinces reporting a vaccination coverage rate higher than 80% by 2019. Hence, greater effects of UVV on the burden of varicella were observed with longer follow-up as target cohorts became eligible for vaccination and vaccination coverage increased. It is important to note that, in Argentina, the one-dose UVV program was implemented for children aged 15 months old without any catch-up program in 2015. Consequently, all 15-month-old cohorts eligible to receive vaccination were progressively added to the target study population of children aged 1–4 years. This caused a gradient effect in the first four years post-UVV (until 2018), when initial and new cohorts of 15-month-olds were progressively added to the target population (1–4 years old). All target cohorts were not fully eligible for vaccination until 2019.

By 2019, observed reductions in incidence rates in peak periods reached highs of 83.9% and 69.1% for target and overall populations, respectively. The reduction in varicella incidence observed in infants aged <12 months and adults could be attributed to the herd immunity effects resulting from a reduction in circulation and exposure to wild-type VZV. In non-peak periods, trends showed sizable differences between observed and predicted incidence rates (−75.4% and −58.3% among target and overall populations, respectively, in 2019). Similar trends were observed for overall periods (combined peak and non-peak periods).

Previous studies have shown the consistent and marked seasonality of varicella in Argentina and other Latin American countries in the pre-UVV era [15,24,35,36]. This seasonality effect was also observed in the current study in the pre-UVV period. However, this study also observed changes in the seasonal trend of varicella incidence in the post-UVV period. The ratio between the highest and lowest incidence months declined over the years as coverage increased and more cohorts were vaccinated in both the target and overall populations. However, a longer follow-up period is needed to better understand the impact of UVV on the seasonality of varicella in Argentina.

Other studies have previously evaluated the impact of UVV in Latin America [22,23,25,37]. The findings of this study are consistent with those reported in a previous ecological study conducted in Argentina from 2008 to 2017. The study showed a declining trend in varicella cases and incidence in Argentina from 2006 with differences becoming more pronounced in 2017, resulting in a 50% reduction in varicella incidence [22]. Similar reductions in incidence were also observed for Brazil and Uruguay following the introduction of UVV, ranging from 38% to 87% [23,25]. Importantly, the magnitude of effects reported across the literature differ based on the time since UVV implementation, dosing schedule, rate of vaccination coverage, and study design.

Notably, varicella vaccination in neighboring Latin American countries may have influenced rates of varicella infection in Argentina, as has previously been seen with other vaccine-preventable diseases. This region has been an example of how regional efforts can stop community transmission, achieving the elimination of diseases such as smallpox, polio, rubella, congenital rubella syndrome, and measles. More recently, the contrary effect was seen with measles, with the reestablishment of endemic transmission after outbreaks in Venezuela, which subsequently reached neighboring countries [38].

Substantial reductions in morbidity and mortality following the implementation of UVV have also been reported in North American and European countries. In the United States, UVV has been implemented for over 25 years and has led to a 92% decrease in varicella incidence and a 90% reduction in deaths across fifteen years [39,40]. In the present study, although a decreasing trend in mortality was observed over time, these differences were not statistically significant. A longer follow-up period is needed to better understand the impact of UVV on mortality in Argentina, to allow comparison with other countries.

Extensive postlicensure experience after distribution of nearly 300 million doses of the live-attenuated Oka/Merck strain continues to show a favorable overall safety profile [41,42]. In Argentina, there is a passive system for reporting adverse events following immunization and for investigating those that are serious. All adverse events associated with vaccines should be reported to the Ministry of Health via their website or by any member of the health care system. From 2015 to 2019, more than 2.5 million doses of the Oka/Merck strain varicella vaccine were given to children in Argentina, with an overall reporting rate of six adverse events per 100,000 doses distributed and less than 0.2 serious adverse events per 100,000 doses, confirming global data with no new safety concerns identified [43].

Although recently published surveillance reports showed reductions in varicella cases in Argentina in 2020, these data may be impacted by multiple factors related to the COVID-19 pandemic [27]. Among them, a decrease in non-COVID vaccine-preventable disease notifications due to limited healthcare workers and resources, social isolation, interruption of face-to-face attendance of children in nurseries and schools, reduction in outpatient consultations for non-COVID diseases, and increased use of hygiene measures [27]. Our study did not include 2020 as the data were not available during the analysis period.

There is still the opportunity to reduce the burden of disease in Argentina through improved vaccination coverage. Although the national coverage reached 81% in 2018, it decreased to 77.6% in 2019 and had a more pronounced decline in 2020 (71.9%) [27,34]. Higher coverage may yield even greater effects. This is particularly important given that routine childhood immunization services have been disrupted worldwide due to the COVID-19 pandemic [44,45,46].

In 2022, Argentina expanded to a two-dose UVV program with the second dose administered at the age of five years and catch-up vaccination for those who were born from October 2013 onwards [27]. This is consistent with the World Health Organization recommendation for the two-dose varicella vaccination to prevent outbreaks and reduce transmission [10]. In other Latin American countries, a second dose addition has been justified by the need for an even higher degree of protection to prevent outbreaks in settings with high contact rates and to reduce severe breakthrough of disease [47,48]. The implementation of two-dose UVV and ongoing efforts to increase vaccination coverage should amplify and accelerate further reductions in varicella burden in Argentina. Continuous epidemiological monitoring is necessary to understand the long-term impact, and additional clinical and economic evaluations are needed to fortify the importance of current vaccination policies.

This study has several limitations. First, the data sources only provided varicella cases that were diagnosed and reported in the official systems. Considering that most cases are mild, some patients may not have sought medical attention, and for those that did, misdiagnosis, miscoding, or misreporting may have also led to an underestimation of varicella incidence. It was assumed that these potential limitations in data reporting were constant over time and did not change with the introduction of UVV. Vaccination coverage was available as aggregated data and not linked to individual patient records; hence, this study could not differentiate between natural versus breakthrough varicella cases. It is also important to highlight certain limitations related to the ecological design of this study. Firstly, the population-level data did not provide additional covariates to evaluate potential confounding factors and their role in the observed results. These covariates could have been impacted by other policies, be the result of underlying secular trends, or be the product of population dynamics impacting host–pathogen interactions, including seasonal changes in host social behavior and contact rates. Changes in these variables over time may have explained part of the observed effect and could not be evaluated. Secondly, it is important to remember that the results of this study focus on the population-level effects of UVV. These can be different from individual-level effects, as is known to occur due to ecological fallacy. Despite these limitations, these data, which were collected as part of notifiable disease reporting over a 12-year period, are assumed to be both sensitive and specific, and represent a real-world scenario in Argentina.

## 5. Conclusions

The findings of this real-world impact study provide evidence of the substantial reductions in the burden of varicella in the target population and also observed indirect effects in the overall population. A substantial decline in varicella incidence was observed within four years of implementation of the one-dose UVV program in Argentina and the impact of UVV increased over time as more cohorts were vaccinated and vaccination coverage increased. This study showed strong seasonality patterns and marked reductions in varicella incidence in peak periods. Continued efforts to increase vaccination coverage rates and epidemiological monitoring will be necessary to understand the long-term impact of the UVV program in Argentina.

## Figures and Tables

**Table 1:** Comparative analysis of varicella cases and incidence pre- and post-UVV introduction, by age group.

Age Groups (Years)	Cases in the Pre-UVV Period								MAIR Pre-UVV	Cases in the Post-UVV Period					MAIR Post-UVV	% Change
	2008	2009	2010	2011	2012	2013	2014	2015-I		2015-II	2016	2017	2018	2019		
<1	12,504	7091	13,627	11,382	8920	11,350	7690	2635	1326.2	6993	5918	4999	3215	3032	823.9	−37.9%
1–4 (target population)	74,485	42,149	82,639	70,075	52,597	69,917	44,474	14,777	1999.1	40,247	34,299	27,220	14,375	12,413	1121.5	−43.9%
5–9	50,145	27,895	60,128	52,957	39,237	60,058	39,341	13,195	1264.3	40,445	40,009	37,354	24,009	23,917	1132.3	−10.4%
10–14	10,368	5766	12,077	10,765	8076	12,180	8421	2960	261.1	9061	10,133	9744	7022	8622	304.9	16.8%
15–24	3807	2197	3756	3301	2455	3253	2138	947	41.0	2111	2476	2220	1864	2238	36.8	−10.4%
25–34	1464	963	1532	1414	956	1220	856	311	17.8	708	904	755	557	596	12.8	−28.4%
35–44	628	387	741	634	459	549	394	162	9.8	333	482	408	324	428	7.7	−21.3%
45–64	471	350	556	487	322	432	333	142	5.1	232	375	303	283	369	4.2	−17.5%
≥65	202	108	274	198	189	188	162	62	4.2	105	193	147	97	211	3.5	−16.3%
Non-targeted population	79,589	44,757	92,691	81,138	60,614	89,230	59,335	20,414	178.0	59,988	60,490	55,930	37,371	39,413	153.6	−13.7%
No age data	12,424	6021	10,181	9997	6075	8587	6237	984		2640	1326	884	287	163	NA	NA
Overall	166,498	92,927	185,511	161,210	119,286	167,734	110,046	36,175	324.9	102,875	96,115	84,034	52,033	51,989	224.1	−31.0%

MAIR, mean annual incidence rate (per 100,000 population); NA, not applicable; pre-UVV, pre-universal varicella vaccination; post-UVV, post-universal varicella vaccination; non-targeted population, all age groups excluding 1–4 years.

## Data Availability

Third Party Data. Restrictions apply to the availability of these data. Data were obtained from the Ministerio de Salud de Argentina (Sistema Integrado de Información Sanitaria Argentino [SISA] and Sistema Nacional de Vigilancia en Salud [SNVS]) and Ministerio de Salud de la Nación (Dirección de Estadísticas e Información de la Salud [DEIS]) and are available at https://sisa.msal.gov.ar/sisa/ (accessed on 12 August 2020) and https://www.argentina.gob.ar/salud/deis/datos (accessed on 12 August 2020) with the permission of the above parties.

## Supplementary Materials

The following supporting information can be downloaded at: https://www.mdpi.com/article/10.3390/vaccines10071151/s1, Figure S1. Trends in mean incidence of varicella by age group over the study period, derived from monthly data; Figure S2. Trends in mean annual mortality rate by age group over the study period; Figure S3. Trends in mean mortality rate by age group over the study period, derived from monthly data; Table S1. Percentage coverage of varicella vaccination in Argentina at the subnational level, 2015–2019; Table S2. Varicella-related deaths by age group over the study period; Table S3. Monthly observed and predicted incidence of varicella and estimated avoided cases in the target population in the post-UVV period; Table S4. Monthly observed and predicted incidence of varicella and estimated avoided cases in the overall population in the post-UVV period; Table S5. Number of observed, predicted, and avoided varicella cases in the target and overall populations post-UVV introduction, considering all months or only months with significant differences; Table S6. Ratios between average incidences in peak versus non-peak periods and highest three versus lowest three incidence months in the target and overall populations, by year.
