# Supplementary material for: Trends in Varicella Burden of Disease Following Introduction of Routine Childhood Varicella Vaccination in Argentina: A 12-Year Time Series Analysis
Source: Vaccines (Basel). 2022 Jul 20;10(7):1151. doi: 10.3390/vaccines10071151 (PMC9317722; doi:10.3390/vaccines10071151)
Supplement: Supplementary file 1 [file vaccines-10-01151-s001.zip › vaccines-1802582-supplementary.pdf]

Supplementary material

Figure S1. Trends in mean incidence of varicella by age group over the study period, derived from monthly data

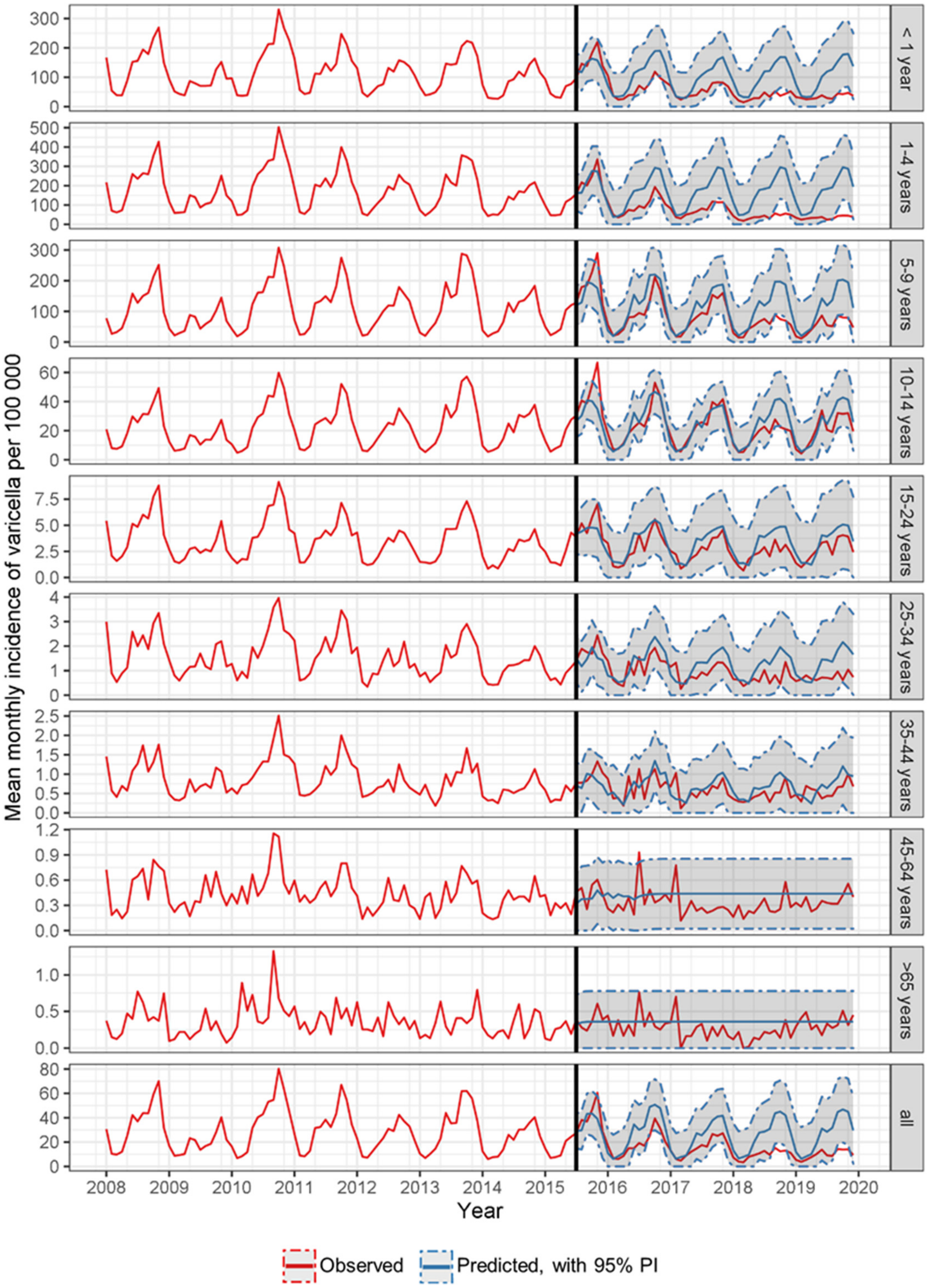

Observed, observed incidence following UVV introduction; predicted, predicted incidence without UVV introduction; PI, predicted interval; UVV, universal varicella vaccination.

Figure S2. Trends in mean annual mortality rate by age group over the study period

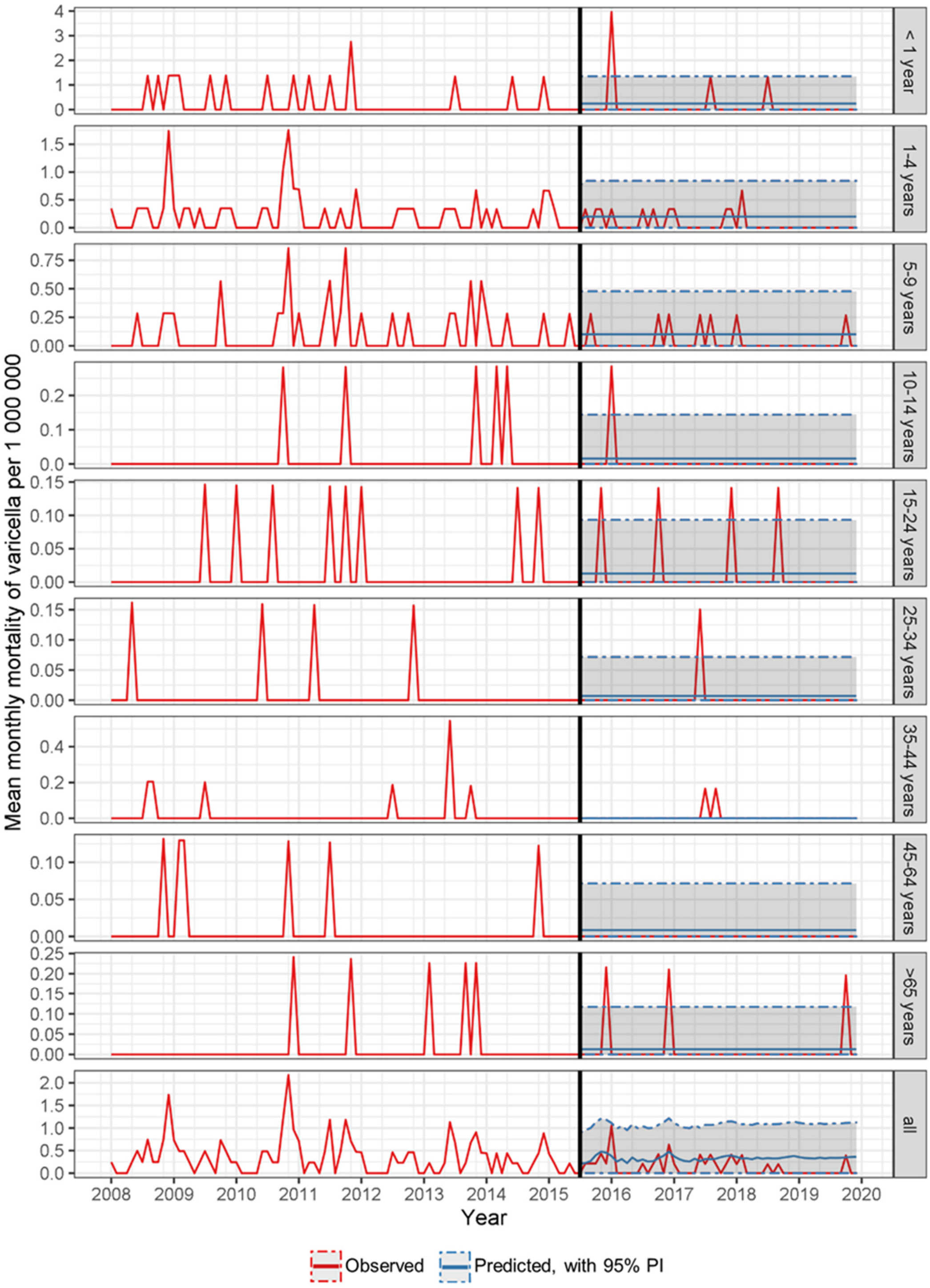

Observed, observed mortality following UVV introduction; predicted, predicted mortality without UVV introduction; PI, prediction interval; UVV, universal varicella vaccination.

Figure S3. Trends in mean mortality rate by age group over the study period, derived from monthly data

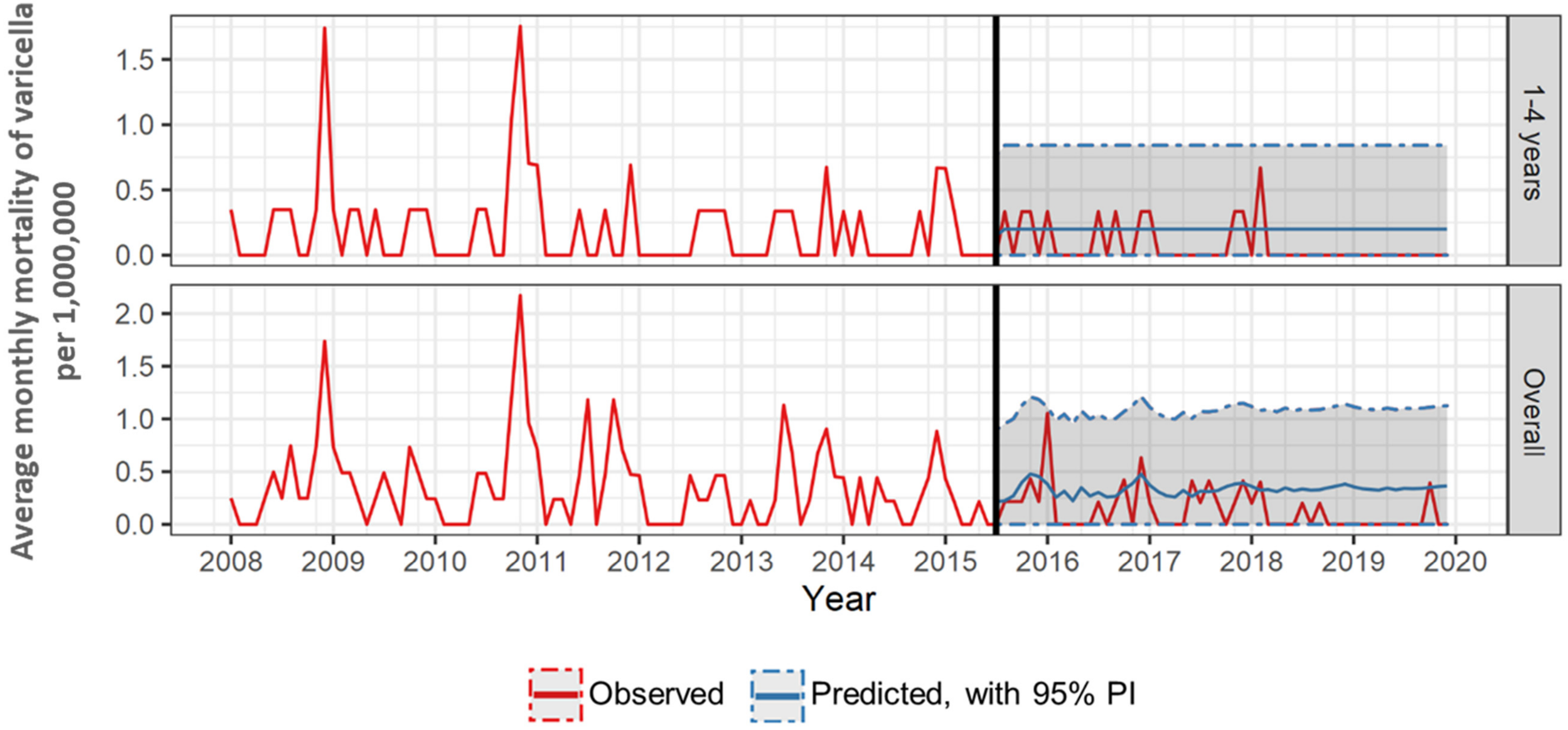

Observed, observed mortality following UVV introduction; predicted, predicted mortality without UVV introduction; PI, prediction interval; UVV, universal varicella vaccination.

Table S1. Percentage coverage of varicella vaccination in Argentina at the subnational level, 2015-2019

Region	2015	2016	2017	2018	2019
Buenos Aires	36.0	69.5	73.7	76.5	69.2
Caba	45.7	71.8	76.0	93.4	89.9
Catamarca	45.0	87.3	76.2	84.3	80.3
Chaco	46.6	77.0	82.5	77.9	82.9
Chubut	51.5	80.9	81.2	86.1	85.8
Cordoba	52.5	79.6	85.5	83.5	82.2
Corrientes	32.7	67.6	62.8	82.7	76.1
Entre Rios	40.0	46.6	53.8	57.2	75.5
Formosa	47.4	77.5	78.0	78.2	82.9
Jujuy	80.9	95.4	89.1	101.8	94.2
La Pampa	54.7	88.3	93.5	90.6	91.2
La Rioja	34.1	72.4	62.5	73.6	65.8
Mendoza	46.7	85.5	87.2	98.9	93.6
Misiones	30.8	62.2	68.9	71.2	77.6
Neuquen	50.5	83.6	90.9	98.5	94.8
Rio Negro	52.2	81.0	74.5	77.9	71.9
Salta	58.7	89.1	82.2	82.7	90.4
San Juan	50.9	69.3	77.8	71.8	79.5
San Luis	92.5	93.6	90.2	94.9	97.8
Santa Cruz	38.4	87.7	83.4	84.5	86.8
Santa Fe	55.1	76.5	76.8	83.7	76.4
Santiago Del Estero	37.0	61.0	91.5	85.8	83.7

Region	2015	2016	2017	2018	2019
Tierra Del Fuego	68.4	83.9	87.6	90.0	89.3
Tucuman	70.5	101.1	82.5	86.9	82.4
National	**44.8**	**74.4**	**76.8**	**81.0**	**77.6**

Vaccination coverage was defined as the total number of immunized children in the target population per total number of target population, based on the national census within the region and period of analysis. In some cases, the vaccination coverage exceeded 100% coverage, due to estimation errors in the population census.

Table S2. Varicella-related deaths by age group over the study period

Age group (years)	Deaths in the pre-UVV period								MAMR Pre-UVV	Deaths in the post-UVV period					MAMR Post-UVV	% Change
	2008	2009	2010	2011	2012	2013	2014	2015-I		2015	2016	2017	2018	2019		
<1	3	4	2	4	0	1	2	0	2.7	0	3	1	1	0	1.3	-51.7%
1–4 (target population)	10	7	12	6	4	5	5	3	2.4	3	4	3	2	0	1.0	-57.7%
5–9	3	3	5	8	3	6	3	1	1.2	1	2	2	1	1	0.4	-62.6%
10–14	0	0	1	1	0	1	2	0	0.2	0	1	0	0	0	0.1	-67.9%
15–24	0	1	2	2	1	0	2	0	0.1	1	1	1	1	0	0.1	-1.7%
25–34	1	0	1	1	1	0	0	0	0.1	0	0	1	0	0	0.0	-62.2%
35–44	2	1	0	0	1	4	0	0	0.2	0	0	2	0	0	0.1	-65.1%
45–64	1	2	1	1	0	0	1	0	0.1	0	0	0	0	0	0.0	-100.0%
≥65	0	0	1	1	0	3	0	0	0.1	1	1	0	0	1	0.2	16.0%
Non-targeted (excluding 1–4 years old)	10	11	13	18	6	15	10	1	0.3	3	8	7	3	2	0.1	-53.9%
All	20	18	25	24	10	20	15	4	0.4	6	12	10	5	2	0.2	-55.7%

MAMR, Mean annual mortality rate (per 1,000,000 population); pre-UVV, pre-universal varicella vaccination; post-UVV, post-universal varicella vaccination; non-targeted population, all age groups excluding age group with 1–4 years old.

Table S3. Monthly observed and predicted incidence of varicella and estimated avoided cases in the target population in the post-UVV period

Model: ARIMA(3,0,1)(0,1,1)[12]								
Year-month	Monthly incidence (x 100,000)				Estimated avoided cases			% Change
	Mean Obs	Mean Pred	Pred – LPI	Pred – UPI	Cases avoided	Low	High	
2015-Jul	158.148	162.806	72.333	253.278	-140	-2861	2581	-2.9%
2015-Aug	217.225	163.177	55.020	271.334	1626	-1628	4879	33.1%
2015-Sep	200.669	225.532	109.656	341.408	-748	-4233	2738	-11.0%
2015-Oct	250.504	276.769	149.617	403.921	-790	-4615	3035	-9.5%
2015-Nov	336.310	273.768	143.519	404.018	1881	-2037	5799	22.8%
2015-Dec	175.170	181.015	45.410	316.620	-176	-4255	3903	-3.2%
2016-Jan	115.184	102.362	0.000	239.626	385	-3734	3456	12.5%
2016-Feb	48.793	38.453	0.000	178.234	310	-3884	1464	26.9%
2016-Mar	35.995	42.739	0.000	183.583	-202	-4428	1080	-15.8%
2016-Apr	47.760	57.825	0.000	199.875	-302	-4564	1433	-17.4%
2016-May	74.589	124.204	0.000	266.875	-1489	-5769	2238	-39.9%
2016-Jun	69.957	172.663	29.397	315.929	-3082	-7380	1217	-59.5%
2016-Jul	94.520	179.273	32.400	326.145	-2543	-6950	1864	-47.3%
2016-Aug	84.888	186.894	38.091	335.696	-3061	-7525	1404	-54.6%
2016-Sep	121.349	240.551	90.700	390.401	-3577	-8073	920	-49.6%
2016-Oct	193.739	292.586	141.487	443.685	-2966	-7499	1568	-33.8%
2016-Nov	152.078	285.644	134.049	437.240	-4008	-8556	541	-46.8%
2016-Dec	104.285	192.146	39.940	344.353	-2636	-7203	1931	-45.7%
2017-Jan	82.390	111.337	0.000	263.722	-867	-5430	2467	-26.0%

Model: ARIMA(3,0,1)(0,1,1)[12]								
Year-month	Monthly incidence (x 100,000)				Estimated avoided cases			% Change
	Mean Obs	Mean Pred	Pred – LPI	Pred – UPI	Cases avoided	Low	High	
2017-Feb	36.703	46.470	0.000	199.184	-292	-4865	1099	-21.0%
2017-Mar	30.959	49.407	0.000	202.290	-552	-5130	927	-37.3%
2017-Apr	50.496	63.657	0.000	216.704	-394	-4977	1512	-20.7%
2017-May	56.775	129.124	0.000	282.266	-2166	-6752	1700	-56.0%
2017-Jun	66.360	176.923	23.702	330.145	-3311	-7898	1277	-62.5%
2017-Jul	81.789	182.893	27.423	338.362	-3027	-7683	1628	-55.3%
2017-Aug	74.943	190.013	33.488	346.538	-3446	-8132	1241	-60.6%
2017-Sep	118.525	243.210	86.135	400.286	-3,733	-8,437	970	-51.3%
**2017-Oct**	**113.950**	**294.872**	**137.064**	**452.679**	**-5,417**	**-10,143**	**-692**	**-61.4%**
**2017-Nov**	**114.752**	**287.597**	**129.546**	**445.647**	**-5,175**	**-9,908**	**-443**	**-60.1%**
2017-Dec	81.422	193.822	35.425	352.219	-3,366	-8,108	1,377	-58.0%
2018-Jan	42.831	112.770	0.000	271.206	-2,090	-6,825	1,280	-62.0%
2018-Feb	23.524	47.699	0.000	206.328	-722	-5,463	703	-50.7%
2018-Mar	18.571	50.458	0.000	209.179	-953	-5,696	555	-63.2%
2018-Apr	28.610	64.558	0.000	223.372	-1,074	-5,820	855	-55.7%
2018-May	36.909	129.896	0.000	288.761	-2,779	-7,527	1,103	-71.6%
2018-Jun	37.243	177.584	18.676	336.493	-4,194	-8,943	555	-79.0%
2018-Jul	44.906	183.459	22.540	344.377	-4,141	-8,950	668	-75.5%
2018-Aug	31.120	190.498	28.654	352.342	-4,763	-9,600	74	-83.7%
**2018-Sep**	**56.785**	**243.625**	**81.307**	**405.944**	**-5,584**	**-10,434**	**-733**	**-76.7%**

Model: ARIMA(3,0,1)(0,1,1)[12]								
Year-month	Monthly incidence (x 100,000)				Estimated avoided cases			% Change
	Mean Obs	Mean Pred	Pred – LPI	Pred – UPI	Cases avoided	Low	High	
**2018-Oct**	**47.014**	**295.227**	**132.258**	**458.196**	**-7,418**	**-12,288**	**-2,547**	**-84.1%**
**2018-Nov**	**56.049**	**287.901**	**124.727**	**451.076**	**-6,929**	**-11,805**	**-2,052**	**-80.5%**
2018-Dec	49.892	194.083	30.604	357.561	-4,309	-9,195	576	-74.3%
2019-Jan	34.412	112.993	0.000	276.493	-2,343	-7,218	1,026	-69.5%
2019-Feb	24.652	47.890	0.000	211.562	-693	-5,573	735	-48.5%
2019-Mar	29.147	50.622	0.000	214.373	-640	-5,522	869	-42.4%
2019-Apr	33.172	64.698	0.000	228.532	-940	-5,825	989	-48.7%
2019-May	34.345	130.016	0.000	293.894	-2,852	-7,738	1,024	-73.6%
2019-Jun	37.699	177.687	13.772	341.602	-4,174	-9,061	713	-78.8%
2019-Jul	24.283	183.547	17.706	349.387	-4,748	-9,693	196	-86.8%
2019-Aug	27.168	190.573	23.848	357.299	-4,872	-9,843	99	-85.7%
**2019-Sep**	**43.267**	**243.690**	**76.513**	**410.867**	**-5,976**	**-10,960**	**-991**	**-82.2%**
**2019-Oct**	**45.280**	**295.283**	**127.482**	**463.083**	**-7,454**	**-12,457**	**-2,451**	**-84.7%**
**2019-Nov**	**44.743**	**287.949**	**119.953**	**455.944**	**-7,251**	**-12,260**	**-2,242**	**-84.5%**
2019-Dec	38.169	194.123	25.837	362.410	-4,650	-9,667	368	-80.3%
				**Averted**	**51,203**			

Blue, highlighted rows signify months where observed incidences following UVV introduction were significantly lower than predicted incidences without UVV introduction.

Apr, April; ARIMA, Autoregressive Integrated Moving Average; Aug, August; Dec, December; Feb, February; Jan, January; Jul, July; Jun, June; LPI: lower prediction interval; Mar, March; Mean Obs, mean observed; Mean Pred: mean predicted; Nov, November; Oct, October; Sept, September; UPI, upper prediction interval; UVV, universal varicella vaccination.

Table S4. Monthly observed and predicted incidence of varicella and estimated avoided cases in the overall population in the post-UVV period

Model: ARIMA(3,0,1)(2,1,0)[12]								
Year-month	Monthly incidence (x 100,000)				Estimated avoided cases			% Change
	Mean obs	Mean Pred	Pred – LPI	Pred – UPI	Cases avoided	Lower	Upper	
2015-Jul	27.467	29.010	14.900	43.120	-666	-6,752	5,420	-5.3%
2015-Aug	38.197	29.748	13.826	45.670	3,644	-3,223	10,512	28.4%
2015-Sep	36.827	43.168	26.481	59.855	-2,735	-9,933	4,462	-14.7%
2015-Oct	46.476	43.460	25.000	61.920	1,301	-6,661	9,263	6.9%
2015-Nov	60.554	39.165	20.534	57.797	9,225	1,189	17,261	54.6%
2015-Dec	28.992	26.336	6.902	45.769	1,146	-7,236	9,528	10.1%
2016-Jan	17.135	10.938	0.000	30.509	2,701	-5,830	7,469	56.7%
2016-Feb	6.740	6.188	0.000	26.024	241	-8,406	2,938	8.9%
2016-Mar	6.050	8.443	0.000	28.403	-1,044	-9,744	2,637	-28.4%
2016-Apr	8.564	11.110	0.000	31.156	-1,110	-9,848	3,733	-22.9%
2016-May	14.992	19.636	0.000	39.763	-2,024	-10,798	6,535	-23.6%
2016-Jun	14.921	34.491	14.328	54.655	-8,531	-17,320	259	-56.7%
2016-Jul	19.231	30.070	9.473	50.667	-4,725	-13,703	4,254	-36.0%
2016-Aug	16.990	32.738	12.000	53.476	-6,864	-15,904	2,175	-48.1%
**2016-Sep**	**25.033**	**48.960**	**28.136**	**69.784**	**-10,430**	**-19,507**	**-1,353**	**-48.9%**
2016-Oct	39.410	50.673	29.722	71.624	-4,909	-14,042	4,223	-22.2%
2016-Nov	31.229	47.901	26.922	68.881	-7,267	-16,412	1,878	-34.8%
2016-Dec	20.202	31.651	10.608	52.694	-4,991	-14,163	4,182	-36.2%
2017-Jan	13.150	12.719	0.000	33.780	190	-9,086	5,792	3.4%

Model: ARIMA(3,0,1)(2,1,0)[12]								
Year-month	Monthly incidence (x 100,000)				Estimated avoided cases			% Change
	Mean obs	Mean Pred	Pred – LPI	Pred – UPI	Cases avoided	Lower	Upper	
2017-Feb	6.089	6.272	0.000	27.358	-81	-9,368	2,682	-2.9%
2017-Mar	4.741	7.726	0.000	28.824	-1,315	-10,608	2,088	-38.6%
2017-Apr	8.955	8.919	0.000	30.027	16	-9,281	3,944	0.4%
2017-May	12.151	16.965	0.000	38.081	-2,120	-11,421	5,352	-28.4%
2017-Jun	14.719	27.275	6.155	48.395	-5,530	-14,832	3,772	-46.0%
2017-Jul	17.543	25.558	3.860	47.257	-3,530	-13,087	6,027	-31.4%
2017-Aug	16.327	30.633	8.768	52.498	-6,301	-15,932	3,329	-46.7%
2017-Sep	26.571	37.785	15.838	59.732	-4,939	-14,606	4,727	-29.7%
2017-Oct	25.208	41.056	18.933	63.178	-6,980	-16,724	2,764	-38.6%
2017-Nov	27.284	41.621	19.476	63.766	-6,315	-16,069	3,439	-34.4%
2017-Dec	18.054	26.724	4.493	48.955	-3,818	-13,610	5,973	-32.4%
2018-Jan	7.333	13.288	0.000	35.536	-2,649	-12,549	3,263	-44.8%
2018-Feb	3.983	6.606	0.000	28.884	-1,167	-11,080	1,772	-39.7%
2018-Mar	3.468	7.881	0.000	30.175	-1,964	-11,883	1,543	-56.0%
2018-Apr	7.886	9.590	0.000	31.894	-758	-10,682	3,509	-17.8%
2018-May	9.228	19.750	0.000	42.063	-4,682	-14,610	4,106	-53.3%
2018-Jun	10.747	27.835	5.517	50.153	-7,603	-17,533	2,327	-61.4%
2018-Jul	12.795	28.591	4.353	52.828	-7,028	-17,813	3,756	-55.2%
2018-Aug	9.579	30.768	6.015	55.522	-9,428	-20,442	1,586	-68.9%
**2018-Sep**	**15.269**	**43.681**	**18.682**	**68.680**	**-12,642**	**-23,765**	**-1,518**	**-65.0%**

Model: ARIMA(3,0,1)(2,1,0)[12]								
Year-month	Monthly incidence (x 100,000)				Estimated avoided cases			% Change
	Mean obs	Mean Pred	Pred – LPI	Pred – UPI	Cases avoided	Lower	Upper	
**2018-Oct**	**12.442**	**44.989**	**19.439**	**70.538**	**-14,481**	**-25,849**	**-3,113**	**-72.3%**
**2018-Nov**	**13.035**	**42.127**	**16.513**	**67.741**	**-12,944**	**-24,341**	**-1,548**	**-69.1%**
2018-Dec	11.177	27.909	2.029	53.788	-7,445	-18,960	4,070	-60.0%
2019-Jan	5.414	11.923	0.000	37.853	-2,925	-14,578	2,433	-54.6%
2019-Feb	3.827	6.297	0.000	32.319	-1,110	-12,804	1,720	-39.2%
2019-Mar	5.456	8.125	0.000	34.191	-1,199	-12,913	2,452	-32.8%
2019-Apr	7.702	10.179	0.000	36.277	-1,113	-12,841	3,461	-24.3%
2019-May	10.192	18.908	0.000	45.034	-3,917	-15,658	4,580	-46.1%
2019-Jun	13.817	31.086	4.946	57.225	-7,761	-19,507	3,986	-55.6%
2019-Jul	9.112	28.495	1.782	55.209	-8,710	-20,715	3,294	-68.0%
2019-Aug	8.654	31.738	4.853	58.624	-10,374	-22,456	1,708	-72.7%
**2019-Sep**	**14.295**	**44.727**	**17.749**	**71.704**	**-13,676**	**-25,799**	**-1,552**	**-68.0%**
**2019-Oct**	**13.843**	**46.794**	**19.644**	**73.943**	**-14,807**	**-27,008**	**-2,607**	**-70.4%**
**2019-Nov**	**13.997**	**44.942**	**17.765**	**72.118**	**-13,906**	**-26,119**	**-1,693**	**-68.9%**
2019-Dec	9.379	29.492	2.230	56.753	-9,038	-21,289	3,213	-68.2%
				**Averted**	**92,886**			

Blue, highlighted rows signify months where observed incidences following UVV introduction were significantly lower than predicted incidences without UVV introduction.

Apr, April; ARIMA, Autoregressive Integrated Moving Average; Aug, August; Dec, December; Feb, February; Jan, January; Jul, July; Jun, June; LPI: lower prediction interval; Mar, March; Mean Obs, mean observed; Mean Pred: mean predicted; Nov, November; Oct, October; Sept, September; UPI, upper prediction interval; UVV, universal varicella vaccination.

Table S5. Number of observed, predicted, and avoided varicella cases in the target and overall populations post-UVV introduction, considering all months or only months with significant differences

			Avoided cases					
			Considering all months			Considering only months with significant differences		
	**Observed**	**Predicted**	**Mean avoided cases**	**Lower**	**Upper**	**Mean avoided cases**	**Lower**	**Upper**
**Target population**	128,328	273,139	-144,811	-391,017	56,484	-51,203	-90,254	-12,152
**Overall population**	387,046	636,136	-249,090	-784,111	186,013	-92,886	-172,388	-13,385

Observed, observed incidence following UVV introduction; predicted, predicted cases without UVV introduction; UVV, universal varicella vaccination.

Table S6. Ratios between average incidences in peak versus non-peak periods and highest three versus lowest three incidence months in the target and overall populations, by year

Year	Target population	Overall population
**Peak period versus non-peak period**		
2015	1.4	1.5
2016	2.1	2.3
2017	1.9	2.1
2018	1.5	1.6
2019	1.4	1.7
**Highest three versus lowest three incidence months**		
2015	5.4	6.1
2016	3.5	4.5
2017	2.9	4.0
2018	2.3	2.7
2019	1.5	2.5

Highest three incidence months, September to November; lowest three incidence months, February to April; target population, children aged 1–4 years.
